# Clinical and hematological profiles of children with dengue residing in a non-endemic zone of Bangladesh

**DOI:** 10.1371/journal.pntd.0010847

**Published:** 2022-10-10

**Authors:** Saiful Islam, Md. Abdullah Saeed Khan, Md. Fakhrul Amin Badal, Muhammad Ziaul Islam Khan, David Gozal, Mohammad Jahid Hasan

**Affiliations:** 1 250 Bedded General Hospital, Tangail, Bangladesh; 2 Pi Research Consultancy Center, Dhaka, Bangladesh; 3 University of Missouri School of Medicine, Columbia, Missouri, United States of America; Army Hospital Research and Referral, INDIA

## Abstract

**Background:**

The clinical and hematological parameters of children with dengue during an outbreak in a non-endemic region have not been well described. To delineate the clinical profile of pediatric cases from a tertiary care center located in a non-endemic zone (Tangail district) in Bangladesh was the objective of the study.

**Methods:**

A cross-sectional observational study was conducted in the Department of Pediatrics of a 250-bed general hospital in Tangail, Bangladesh, between June 2019 to September 2019. Data collection was done using a pre-structured case record form. All patients underwent detailed history taking, physical examination, and hematological profiling. A total of 123 confirmed dengue cases were analyzed.

**Results:**

The average age of patients was 7.3±4.1 (SD) years, with nearly two-thirds being male (61.8%) and the majority living in rural areas (76.4%). Fever (100%), body ache (57.7%), headache (56.9%), and rash (55.3%) were the four common clinical manifestations. NS1 antigen and anti-dengue IgM antibody tests were positive in 86% (102 out of 119) and 37.7% (20 out of 53) of cases, respectively. Thrombocytopenia was present in 42% of cases. The majority of the cases had dengue fever (73.2%), and the remaining cases were either dengue hemorrhagic fever or dengue shock syndrome (26.8%). Clinical and hematological parameters varied with the type of dengue. Particularly, rash (p = <0.001), bleeding manifestation (p = <0.001), vomiting (p = 0.012), hypotension (p = 0.018), pleural effusion (p = 0.018), ascites (p = 0.018), hepatomegaly (p = <0.001) and low platelet count (<150 x 10^3^cells/μL) (p = 0.038) were significantly more common among dengue hemorrhagic fever or dengue shock syndrome cases.

**Conclusions:**

The present study documented the clinical features of dengue in a pediatric group of patients from a non-endemic zone of Bangladesh. This vulnerable patient group requires earlier identification and keen attention during management.

## Author summary

Bangladesh saw an increasing frequency of dengue outbreaks over the last two decades. Most of the time, it was confined to large cities endemic to the disease. However, in 2019 a massive dengue outbreak occurred in the country, which spread from endemic to non-endemic regions of the country. The present study documented clinical and hematological profiles of 123 children with dengue who got admitted between June 2019 to September 2019 to a 250-bed general hospital in Tangail, a non-endemic region. Patients had an average age of just over seven years, and nearly two-thirds were male. More than one-quarter of patients had dengue fever with bleeding manifestation (dengue hemorrhagic fever). Two of the children suffered from dengue shock syndrome. Fever, body ache, headache, and rash were predominant clinical features. Thrombocytopenia was present in two-fifth of the cases. The presence of dengue in a non-endemic zone warrants further epidemiological, entomological, and environmental research in the area.

## Introduction

Dengue is the most common arboviral infection transmitted to humans by the bite of mosquito vectors *Aedes aegypti* and *Aedes albopictus*. [1] Approximately 100 million dengue infections are estimated to occur annually, with almost 10,000 deaths being reported in 125 countries worldwide. [2,3] In Bangladesh, the first recorded epidemic occurred in the mid-2000s when 5,551 dengue infections were identified, and the case fatality rate was 1.6%, with 93 deaths. [4] The 2019 dengue outbreak in Bangladesh was a nationwide epidemic that began during early Spring in Dhaka and then spread throughout the country during the Eid festival when people traveled from Dhaka city to their village-homes. [5] By the end of the year, the officially reported number of hospitalized patients exceeded 100,000 cases with 129 deaths, [6] with the actual number of cases being estimated as much higher.

Patients suffering from dengue may be asymptomatic or may present with a wide range of clinical manifestations. [7] Symptomatic dengue patients can develop a wide spectrum of disease severity ranging from an influenza-like illness (dengue fever-DF) to dengue with bleeding manifestations (dengue hemorrhagic fever-DHF), a proportion of which may exhibit a disseminated vascular leakage ultimately developing shock (dengue shock syndrome-DSS). Infants and children are more susceptible to develop DSS in comparison with adults. [8] Infection with dengue is usually suspected by clinical manifestations and confirmed by hematological and microbiological laboratory testing, i.e., viral antigen detection, specific serology, or virus isolation in cell cultures. [9]

The pattern of infection, clinical manifestations, and severity showed spatiotemporal variation across studies conducted over the last twenty years in Bangladesh. [4,10–15] So far, the outbreaks were primarily restricted to major cities, and a nationwide seroprevalence study found that districts in the north half of the countries were relatively spared. [16] During the 2019 outbreak, dengue cases rapidly spread across all regions of the country [17] including non-endemic zones, where a less severe primary infection would be theoretically expected, and the clinical characteristics of such cases could potentially aid in setting up strategies for early detection, stratification, and management of patients aimed at minimizing the risk of more severe disease. Notwithstanding, studies detailing the clinical features and hematological profiles of dengue cases in children are scarce, and were reported primarily from the capital city of Bangladesh, Dhaka city. [14,15] Therefore, the objective of this study was to describe the clinical profile of pediatric cases from a secondary care center located in a non-endemic zone (Tangail district) in Bangladesh.

## Methods

### Ethical statement

The study protocol was reviewed and approved by the Ethical Review Committee (ERC) of Tangail General Hospital. Informed written consent was obtained from the parents/guardians of all the children. In addition, informed assent was taken from children ≥ 14 years. The current guidelines of World Medical Association Declaration of Helsinki was followed during the study work.

### Study design and setting

This cross-sectional observational study was conducted in the Department of Pediatrics in a 250-bed general hospital in Tangail, Bangladesh, during the period from June 2019 to September 2019.

### Study population

All children (n = 152) who were admitted to the designated dengue ward within the study period with clinical features suggestive of dengue were approached, and 129 children whose serological test was positive for dengue (either or both of NS1Ag and anti-dengue IgM antibody) were considered for inclusion in the study. However, six children were excluded from the final analysis due to incomplete data. Finally, a total of 123 children were included.

### Case definition

Dengue cases were defined and classified according to ‘Pocket Guideline for Dengue Case Management 2019’ published by DGHS, Bangladesh. [18] and ‘Comprehensive guidelines for prevention and control of dengue and dengue hemorrhagic fever’ revised and expanded edition by the WHO [19]. Patients were categorized into following three classes- dengue fever (DF, including classical dengue and dengue with unusual hemorrhage), dengue hemorrhagic fever (DHF), and dengue shock syndrome (DSS). Primary infection was defined as having positive IgM antibody and negative IgG antibody or having an IgM:IgG ratio of >1.8 at day 7 after the onset of illness. However, having a negative IgM and positive IgG test or an IgM:IgG ratio of ≤1.8 was considered secondary infection.

### Data collection and data processing

A structured questionnaire was used for data collection and this instrument was validated through previous piloting. Each patient underwent a detailed history taking, and a thorough physical examination, and a blood draw was obtained for measurement of hemoglobin, total leucocyte count (TLC) and differential leucocyte count (DLC), platelet count, and hematocrit (HCT). Blood cultures, Weil-Felix test, chest x-ray and other investigations were performed as clinically dictated by patient’s condition to determine co-infections. All study cases were routinely followed up daily until discharge.

### Statistical analysis

Statistical analysis was conducted using SPSS 26 software for Windows (IBM, Armonk, NY). The regional distribution map was created using ArcGIS Desktop 10.5 (Esri Inc., Redlands, CA). Descriptive statistics were used for continuous variables, while categorical variables were expressed as frequency and percentages. Comparison of clinical characteristics and hematological profiles between the children with DF and DHF was performed using Chi (χ)-square or Fisher’s exact test, independent samples t test, and Mann-Whitney U test, as appropriate.

## Results

### Demographic characteristics and comorbidity profile

**Fig 1:**
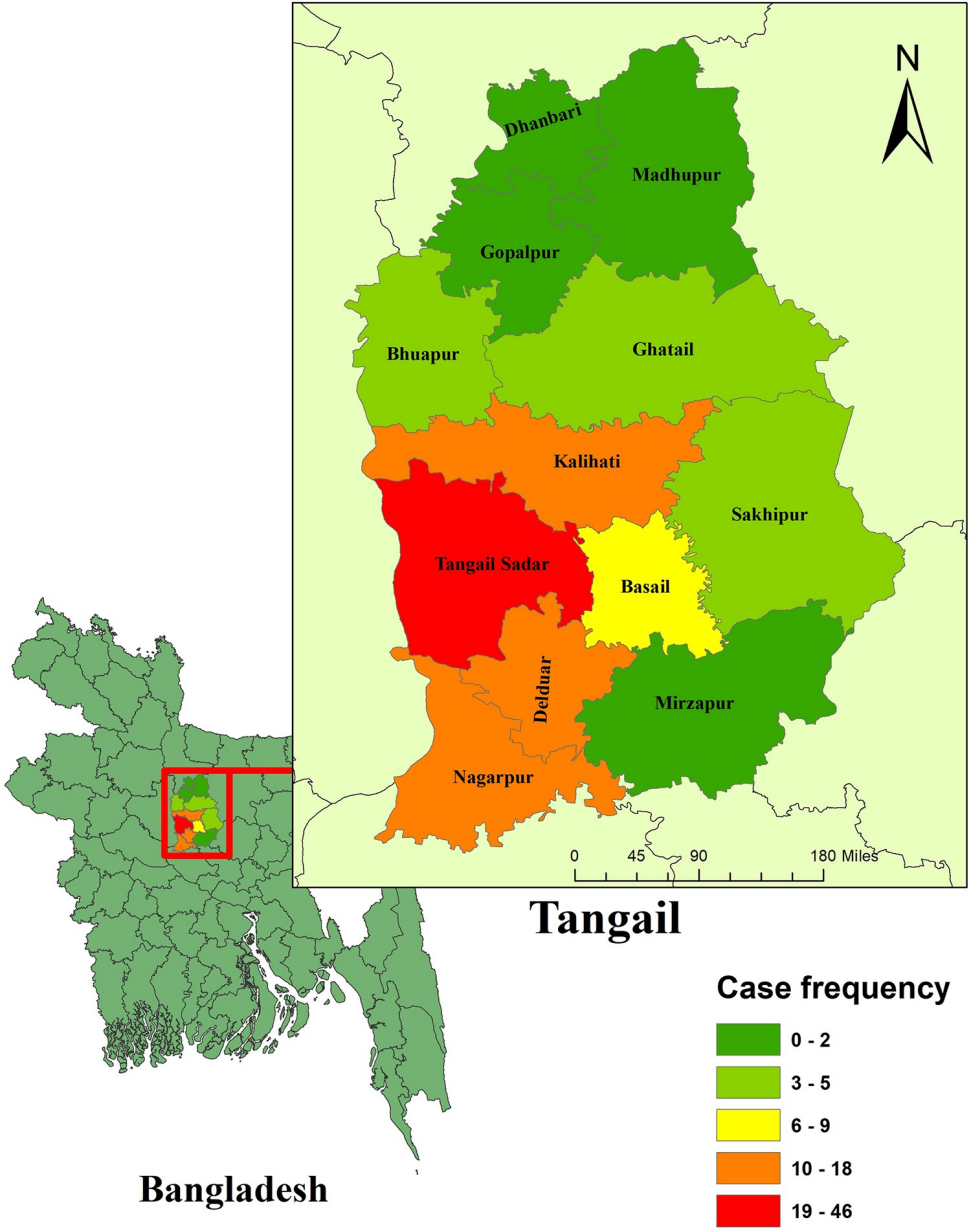
Regional distribution of children with dengue in the non-endemic region of Tangail in Bangladesh (n = 123) [The shapefile of the base layer used in this map is freely available from https://data.humdata.org/dataset/cod-ab-bgd, under Creative Commons Attribution 4.0 International License (https://data.humdata.org/faqs/terms)].

A total of 123 children with dengue were included in the final anlaysis. These were primarily concentrated in four sub-districts, namely Tangail Sadar, Kalihati, Delduar, and Nagarpur **(Fig 1)**.

**Fig 2:**
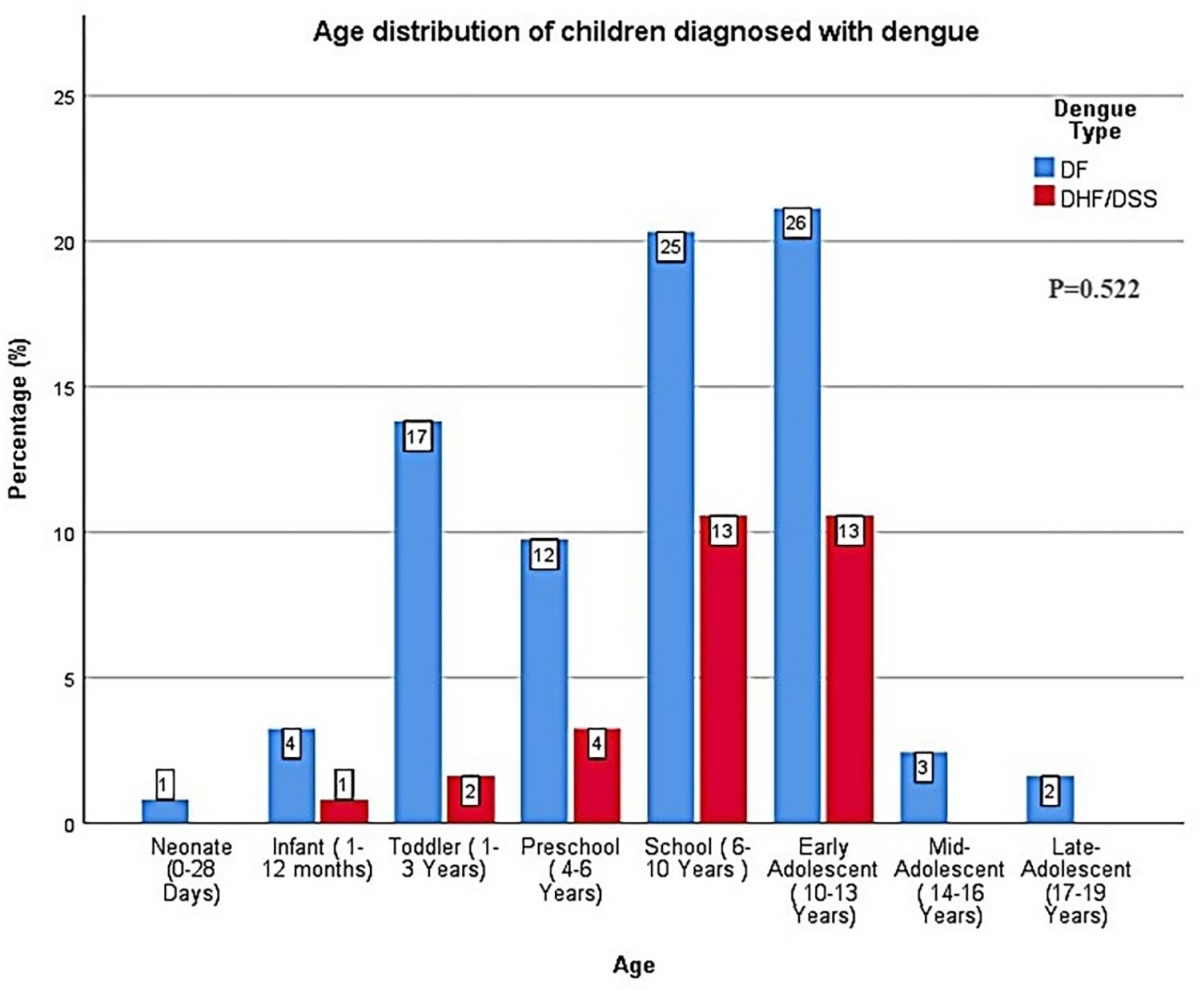
Age distribution of children with dengue (The bars represent percentage and the data within boxes show frequency; p-value was determined by Fisher’s exact test).

The mean age of the subjects was 7.3±4.1 (SD) years (ranging from 5 days to 17 years), with school-age (6–10 yrs.) and early adolescents (11–13 yrs.) comprising more than half of the cases (62.6%). DHF was more common in the mid-age range, with relative sparing of neonates, and mid- or late-adolescent children. However, there were no significant association between patient age and dengue severity types (**Fig 2**), with 90 of the cases (73.2%) presenting with DF. Among the rest (26.8%), 31 cases had DHF and 2 had DSS. A detailed presentation of the DSS cases is provided in **S1 Table**. The dengue infected children were predominantly male (61.8%) and were residing mostly in rural areas (76.4%). Most of the children had no history of travel to an endemic zone within 2 weeks of onset of symptoms (88.6%). DF and DHF/DSS did not show any significant differences regarding sex, residence and history of travel to endemic zones. However, typhus fever was significantly associated with DHF/DSS (p = 0.044) compared to DF. (**Table 1**).

**Table 1:** Demographic, serological and co-morbidity profile of children with dengue.

Characteristics	Total	DF	DHF/DSS	P value
Total patients	123	90 (73.2)	33 (26.8)	
**Sex (Male)**	76 (61.8)	58 (76.3)	18 (23.7)	0.317
**Residence**				
Rural area	94 (76.4)	65 (69.1)	29 (30.9)	0.221
Semi-urban area	16 (13.0)	14 (87.5)	2 (12.5)	
Urban area	13 (10.6)	11 (84.6)	2 (15.4)	
**H/o travel to endemic zone within last two weeks**	14 (11.4)	11 (78.6)	3 (21.4)	0.870
**Comorbidities**				
Enteric fever	7 (5.7)	3 (42.9)	4 (57.1)	0.154
Typhus	6 (4.9)	2 (33.3)	4 (66.7)	**0.044**
Tonsillitis	6 (4.9)	5 (83.3)	1 (16.7)	0.488
Pneumonia	3 (2.4)	2 (66.7)	1 (33.3)	0.612
UTI	3 (2.4)	2 (66.7)	1 (33.3)	0.612
NS1 Antigen (present)* (*Missing values = 4)*	102 (85.7)	77 (75.5)	25 (24.5)	0.255
Anti-Dengue IgM antibody (present)* (*Missing values = 70)*	20 (37.7)	14 (70.0)	6 (30.0)	0.635
Anti-Dengue IgG antibody (present)* (*Missing values = 71)*	6 (11.5)	4 (66.7)	2 (33.3)	0.608
Type of infection* (*Missing values = 71)*				
Primary	46 (88.5)	32 (69.6)	14 (30.4)	1.000
Secondary	6 (11.5)	4 (66.7)	2 (33.3)	

Data are expressed as n (%)

The numbers within parenthesis express percentage within column for total and percentage within row for dengue classes.

*percentage was calculated based on available data

p value determined by Chi (χ)-square test, Yate’s continuity correction, and Fisher’s exact test, as appropriate

### Serological features

Among the cohort, all of NS1, IgM and IgG tests reports were available in 52 cases. Among those cases 46 (88.5%) had primary infection and rest (11.5%) had secondary infection. NS1 antigen was present in 85.7% of the cases, and IgM antibody was detected in 37.7% cases. Only 6 out of 52 cases (11.5%) showed positivity for IgG antibody. **(Table 1)**

### Clinical features

The major presenting complaints other than fever (median duration of 6 days) were myalgia, headache, skin rash, vomiting, abdominal pain, diarrhea, and retro-orbital pain. At least 37 (30.1%) of the children presented with mucosal bleeding manifestations, with the majority (64.9%) having melena. Other less frequent symptoms included dyspnea, lethargy, oliguria, and various neurological manifestations. Two child developed shock. There were no significant differences in the mean duration of fever and hospital stay between children with DF and DHF/DSS. Compared to children with DF, children with DHF/DSS were significantly more likely to vomit, bleed, and develop skin rash, hepatomegaly, and signs of plasma leakage (including hypotension, pleural effusion, and ascites) (**Table 2**).

**Table 2:** Clinical features of children with dengue at presentation.

Characteristics	Total (n = 123)	DF (n = 90)	DHF/DSS (n = 33)	P value
**Duration of fever (days)**	6 (2–22)	5 (2–20)	6 (3–22)	0.103
**Duration of hospital stay (days)**	5 (2–11)	5 (2–11)	6 (3–11)	0.191
***Symptoms***				
Fever	123 (100)	90 (100)	33 (100)	1.000
Headache	70 (56.9)	46 (51.1)	24 (72.7)	0.079
Body ache	71 (57.7)	48 (53.3)	23 (69.7)	0.206
Retro-orbital pain	26 (21.1)	17 (18.9)	9 (27.3)	0.383
Lethargy	10 (8.1)	8 (8.9)	2 (6.1)	0.611
Rash	68 (55.3)	41 (45.6)	27 (81.8)	**<0.001**
Flushing	59 (86.8)	38 (92.7)	21 (77.8)	
Maculopapular	7 (10.3)	3 (7.3)	4 (14.8)	
Petechiae or purpura	2 (2.9)	0	2 (7.4)	
Bleeding	37 (30.1)	5 (5.6)	32 (97.0)	**<0.001**
Malena*	24 (64.9)	3 (60.0)	21 (65.6)	
Hematemesis	3 (8.1)	0	3 (9.4)	
Epistaxis	3 (8.1)	0	3 (9.4)	
Skin bleeding	2 (5.4)	1 (20.0)	1 (3.1)	
Per vaginal bleeding	2 (5.4)	1 (20.0)	1 (3.1)	
Hematuria	1 (2.7)	0	1 (3.1)	
Bleeding from multiple site	2 (5.4)	0	2 (6.3)	
Abdominal pain	36 (29.3)	23 (25.6)	13 (39.4)	0.237
Vomiting	45 (36.6)	27 (30.0)	18 (54.5)	**0.012**
Diarrhea	24 (19.5)	17 (18.9)	7 (21.2)	0.773
Dyspnoea	11 (8.9)	8 (8.9)	3 (9.1)	0.972
Oligouria	6 (4.9)	6 (6.7)	0	0.190
Neurological symptoms	3 (2.4)	3 (3.3)	0	0.563
Shock	2 (1.6)	0	2 (6.1)	0.070
***Signs***				
Temperature (F)	102 ±1.5	102 ±1.3	102 ±1.9	0.632
Tourniquet test (positive)	1 (0.8)	0	1 (3.0)	0.165
Hypotension	3 (2.4)	0	3 (9.1)	**0.018**
Pleural effusion	3 (2.4)	0	3 (9.1)	**0.018**
Ascites	5 (4.1)	1 (1.1)	4 (12.1)	**0.018**
Hepatomegaly	20 (16.3)	8 (8.9)	12 (36.4)	**<0.001**
Splenomegaly	9 (7.3)	4 (12.1)	5 (5.6)	0.215

Data is expressed as n (%), median (range) and mean ±SD.

p value determined by χ-square test, Fischer’s Exact test, Mann-Whitney U test and independent samples t test where appropriate.

*Blood mixed stool

### Hematological profiles

Mean hemoglobin levels were within the normal range (12.1±1.5 g/dl). Thrombocytopenia (platelet count ≤ 150 x10^3^cells/μL) was the most common (42.3%) hematological abnormality, followed by leukopenia (total leucocyte count < 4 x 10^3^ cells/μL) (28.5%). An increased hematocrit (> 48%) was identified in 3.3% of cases. Thrombocytopenia was significantly more common (p = 0.038) in children with DHF compared to children with DF, and consequently mean platelet count was significantly lower (p = 0.040). Other hematological parameters were statistically similar across groups. **(Table 3)**

**Table 3:** Hematological profile of children with dengue at admission.

Variable	Total (n = 123)	DF (n = 91)	DHF/DSS (n = 33)	P value
Hemoglobin (g/dl)	12.1 ±1.5	12.1 ±1.4	12.1 ±1.7	0.988
Hematocrit (%)	36.4 ±4.7	36.3 ±4.5	36.6 ±5.3	0.811
Hematocrit >48 (raised hematocrit)	4 (3.3)	2 (2.2)	2 (6.1)	0.292
Leucocyte (x 10^3^ cells/μL)	6.2 ±3.3	6.5 ± 3.6	5.6 ±2.6	0.215
Leucocyte <4 (leucopenia)	34 (27.6)	23 (25.6)	11 (33.3)	0.393
Platelet (x 10^3^ cells/μL)	168.3 ±82.1	177.5 ±82.1	142.9 ±77.9	**0.040**
Platelet <150 (thrombocytopenia)	52 (42.3)	33 (36.7)	19 (57.6)	**0.038**

Data is expressed as n (%) and mean ±SD

p-value was determined by independent samples t test and Chi-squared test or Fisher’s exact test where appropriate.

## Discussion

The present study is the first to report the clinical and hematological characteristics of dengue in a pediatric cohort residing in a non-endemic area of Bangladesh during an outbreak. The vast majority presented as DF and the rest had DHF/DSS, with only two cases of shock. This disease pattern markedly differs from studies conducted in Dhaka city, where more than one-fourth of all affected children developed DSS during an outbreak. [14,15] All other features of the disease were aligned with the differential phenotypic distribution of the disease in this non endemic region,

In a previous study, a nationwide seroprevalence assessment by Salje and colleagues showed that Dhaka city along with Chittagong and Khulna cities had a high frequency of past dengue infection, while communities in the northern half of the country were largely unaffected. [16] On the other hand, it is now well-established that a secondary infection by a different virus strain can lead to severe dengue through antibody-dependent enhancement. [20] Since the prevalence of secondary infection and history of travel to endemic zones were infrequent in our study, these might explain why a higher proportion of shock did not occur. Another interesting finding was a high prevalence of dengue among children coming from the rural areas, which implies a rural expansion of the 2019 outbreak in Bangladesh, and supports the evidence of recent rural expansion of the virus in the South East Asian countries. [21]

We noted typical clinical presentations of dengue in our study participants with myalgia (in the form of either backache and diffuse pain), headache and rash in more than half the patients, and gastrointestinal features and bleeding manifestations in more than one-quarter of the patients. Previous reports from Bangladesh in 2018 [14,15] and from Papua New Guinea in 2016 [22] reported lower frequency of such symptoms. However, a study from Indonesia that included children with dengue between 2009 to 2013 reported a similar prevalence of pain symptoms. [23] Also, the predominant bleeding manifestation in our study was melena, while epistaxis was the most common bleeding presentation noted in the studies conducted in Bangladesh a year earlier. [14,15] Intriguingly, during the 2000 outbreak in Dhaka city, the pattern of clinical picture was remarkably similar to our findings. [10] A study conducted among adult patients in Bogra, another non-endemic zone for dengue during the 2019 outbreak, noted a similar clinical pattern where pain symptoms were more common and gastrointestinal warning signs and bleeding manifestations were less frequent. [24] By definition bleeding, rash, and signs of plasma leakage was present in a significantly higher proportion in the DHF/DSS group. Similar to Ramabhatta *et al*., [25] a significantly higher proportion of children with DHF had hepatomegaly compared to DF fever patients. It is commonly accepted that all of these features are warning signs for the development of severe disease, [7] and, therefore, the previous and current low frequency of warning signs such as abdominal pain, vomiting, bleeding, and signs of plasma leakage [26] seem to indicate that the first outbreak in a non-endemic zone might be less severe than subsequent outbreaks.

The hematological findings of our patients resemble those of Mishra *et al*. [27] in that they didn’t find any significant difference between non-severe and severe dengue in terms of leucocyte count and hematocrit values. But unlike these authors we noted a significantly higher proportion of children with DHF presenting with thrombocytopenia, a finding that is also supported by Yolanda and Alfan. [28] Therefore, presence of thrombocytopenia should warrant a suspicion of severity and should be addressed promptly.

We noted a predominance of older and male children among the infected cases, which corresponds to the recent epidemiological shift that has become evident in South-East Asian regions. [21] In order to explore sex-related differences in the prevalence of dengue in more detail, Anker and Arima [29] reviewed the literature, and noted that the difference is small and is not consistently present in pediatric patients. However, as most of the children came from relatively less developed rural areas in our study, a predominance of male children seeking healthcare services may reflect cultural issues rather than any other biological factors. [27,30]

Our study adds to the body of knowledge regarding the clinical and hematological characteristics of pediatric dengue in a non-endemic area. However, the study was limited in that this was a single center study, the sample size was relatively small, and that liver function tests and ultrasonography of hepatobiliary system could not be evaluated in all patients. Nevertheless, our findings provide a strong rationale for pursuing predictors of dengue severity among children from non-endemic zones.

## Conclusion

The first-ever recorded dengue outbreak among children in a non-endemic area was characterized by less severe disease, with only one-fourth developing DHF and only two children developing DSS. The predominant clinical manifestations alongside fever were pain and the presence of a rash, and children were more likely to be male and originate from rural areas. Thrombocytopenia was the most common hematological abnormality. Further exploration of possible causes of dengue in a non-endemic region and potential predictors of dengue severity in children warrant future epidemiological, entomological and environmental research in this area.

## Supporting information

S1 Checklist STROBE checklist. (DOCX)

S1 Table Detail of the presentation of dengue shock syndrome (DSS) cases. (DOCX)
