# Supplementary material for: Clinical and hematological profiles of children with dengue residing in a non-endemic zone of Bangladesh
Source: PLoS Negl Trop Dis. 2022 Oct 10;16(10):e0010847. doi: 10.1371/journal.pntd.0010847 (PMC9584401; doi:10.1371/journal.pntd.0010847)
Supplement: S1 Table — (DOCX) [file pntd.0010847.s002.docx]

**S1 Table.** **Detail of the presentation of dengue shock syndrome (DSS) cases**

| **Demographic profile** | **Case 1** | **Case 2** |
| --- | --- | --- |
| Age | 10 years | 13 years |
| Sex | Male | Female |
| **Comorbidity** | None | None |
| **Clinical features** |  |  |
| Fever | Present | Present |
| Duration of fever before admission | 5 days | 6 days |
| Headache | Present | Present |
| Body ache | Absent | Present |
| Retro-orbital pain | Absent | Absent |
| Bleeding manifestation | Malena | Hematuria |
| Abdominal pain | Absent | Present |
| Vomiting | Absent | Present |
| Shock | Present | Present |
| Weakness | Present | Present |
| Highest recorded temperature at admission | 102 | 100 |
| Blood pressure at admission in mmHg (SBP/DBP) | 80/60 | 80/50 |
| Tourniquet test | Negative | Negative |
| Hypotension | Present | Present |
| Ascites | Present | Present |
| Pleural effusion | Present | Absent |
| Hepatomegaly | Present | Absent |
| Splenomegaly | Present | Absent |
| **Hematological profile** |  |  |
| Hemoglobin (g/dl) | 16.90 | 13.00 |
| Hematocrit (%) | 53% | 41% |
| WBC count (/mm^3^) | 2700 | 4890 |
| Platelet count (/mm^3^) | 35000 | 25000 |
| NS1 Antigen | Positive | Positive |
| NS1 done (how many days after onset of fever) | 3 days | 5 days |
| IgM /IgG | Not done | Not done |
